# Client perceived quality of the postnatal care provided by public sector specialized care institutions following a normal vaginal delivery in Sri Lanka: a cross sectional study

**DOI:** 10.1186/s12884-019-2645-4

**Published:** 2019-12-09

**Authors:** Sashimali Anuradha Wickramasinghe, Moraendage Wasantha Gunathunga, Dewabandu Kumarathungalage Nilmini Nilangani Hemachandra

**Affiliations:** 1 Ministry of Health, Nutrition and Indigenous Medicine, Colombo 10, Sri Lanka; 2 Department of Community Medicine, Faculty of Medicine, University of Colombo, Colombo, Sri Lanka; 3 WHO Regional Office for the Eastern Mediterranean, Cairo, Egypt

**Keywords:** Client perceptions, Postnatal care, Quality of care, Client perceived care, Institutional postnatal care

## Abstract

**Background:**

Majority of the maternal and neonatal adverse events take place during the postnatal period. Provision of high-quality care during this period can minimize these events. Assessment of mothers’ perceptions of the quality of care received by them provides valuable feedback to improve the care and ultimately outcomes.

**Methods:**

A cross sectional survey was conducted in specialized institutions of Colombo district, Sri Lanka, to assess the maternal perceptions of the quality of regular postnatal care and its correlations, using an interviewer administered questionnaire. The questionnaire contained 23 items distributed under three main domains: technical and information domain, interpersonal care domain and ward facilities and cleanliness domain. Each item was given a score from 1 to 5 and total scores were calculated for the total questionnaire and for each domain. Descriptive statistics were used to assess the perceptions and multivariate analysis was conducted to assess the significant correlates of positive perceptions.

**Results:**

The median score obtained for the questionnaire was 108, (Inter Quartile Range 96–114). The median scores of the technical care and information domain, interpersonal care domain and ward facilities and cleanliness domain were 43 (IQR 38–45), 33 (IQR 30–35) and 32 (IQR 28–35) respectively. Attending teaching/ specialized hospitals (aOR=1.6, *p* < 0.001), 20–35 age group (1.8, *p* = 0.024), and services such as initiation of breast feeding within 1 h of delivery (2.1, *p* = 0.009), pain relief during episiotomy suturing (2.2, *p* < 0.001), practicing Kangaroo Mother Care (1.4, *p* = 0.035), receiving health advices by doctors or midwives (2.1, *p* < 0.001) were significant correlates of positive perceptions.

**Conclusions:**

Majority of mothers had favourable perceptions of the quality of care received by them. However, the ward facilities and environment domain has obtained lower ratings compared to technical and interpersonal care domains. Several services were significantly associated with favourable perceptions. Authorities should consider these findings when attempting to improve care quality. Further, this assessment should be carried out regularly to obtain more current data.

## Background

The first few weeks following the delivery of a baby is a crucial period for both mother and the newborn as a majority of maternal and newborn adverse outcomes takes place in this period [1–3]. Immediate postnatal period or the first 24 h after delivery is the most vital period, where more than half of the postpartum maternal deaths [4] and around 40% of the newborn deaths occur [5]. Provision of evidence-based care with adequate quality during this period is vital to ensure a smooth recovery of the mother and the baby.

Quality of health care is now considered an essential component of health services [6]. It ensures that services are effective, efficient, patient centered, cost effective and safe [4]. Further, it should be regularly monitored and upgraded to ensure best outcomes. Assessment of client experiences and perceptions of the care is increasingly being considered as a useful monitoring measure [7–10]. Client perceived quality is defined as “subjective and dynamic perception of the extent to which expected health care is received by a person” [11]. It provides health workers and authorities with valuable information to improve the service quality and render it patient-centered. Client perceptions of the quality of service received by them will also determine their level of satisfaction with the services. It is known that unsatisfied clients may not return to the same facility even during an emergency and even if the facility provides state of the art care [6, 10].

In Sri Lanka, around 99.9% women receive institutional postnatal care, and a clear majority of them (94.6%) receive care from public sector institutions [12]. Evidence based practices are implemented in these institutions through national guidelines, and regular supervision is aimed to maintain the highest level of care. However, client perceived quality is not a popular measure of quality in this context. Further, though studies have assessed mothers’ perceptions and satisfaction of antenatal and intra-natal services [13, 14], studies on institutional postnatal care as sparse in Sri Lanka.

Therefore, this study was conducted with the aim of assessing the quality of the regular postnatal care provided by the public sector specialized institutions in the district of Colombo, Sri Lanka, following a normal vaginal delivery (NVD), from the mothers’ perspective, and the factors that are associated with their perceptions. This is a component of a larger study that evaluated the quality of the regular institutional postnatal care comprehensively. We hope that the information gained from this study would be beneficial to improve the institutional postnatal care services in Sri Lanka to meet mothers’ expectations.

## Methods

A cross sectional analytical study was conducted from the 1st December 2016 to the 30th April 2017 in the public sector specialized health institutions in Colombo district. Colombo District is situated in the south west of Sri Lanka and has an area of 699 km^2^. It is a mixture of urban, semi urban, rural and estate areas. It has the highest population in Sri Lanka, which is 20,359,439 [15].

### Study setting

Curative health sector in Sri Lanka is comprised of three levels: primary, secondary and tertiary. Primary health services provide the field and the first contact care, while the secondary and tertiary level institutions provide specialized services under the guidance of specialist health professionals. Thus, in the institutions providing specialized Maternal and Newborn Health (MNH) care, both routine basic care and comprehensive care for emergencies and complications are provided through a team of trained health care providers.

Of the 330,898 deliveries that that took place in government hospitals in 2014, the highest proportion (12.6%) took place in Colombo district [12]. It is worthwhile to note that Colombo district also reports the lowest rate of vaginal deliveries, which was 55.1% in year 2017 [16]. This may be attributed to the fact that the two main referral institutions for pregnant mothers are situated in this district.

The six state sector health care institutions providing specialized MNH care in Colombo account for 98.4% of the total deliveries [17]. These six institutions include three base hospitals (secondary level), one teaching hospital (tertiary level) and two special hospitals specialized to provide women’s’ health services only. Altogether, there are 17 wards providing specialized MNH services in these six institutions.

### Study population and sampling

The study population consisted of mothers who had been discharged following a normal vaginal delivery. Mothers who had complications and the mothers with newborns who had complications following a normal delivery were excluded from the study as their care would deviate from the regular postnatal care, which was the focus of the study. A sample of 1300 mothers was deemed necessary to detect the significant associations of client perceptions.

Mothers were selected from all 17 wards. Number of mothers selected from each ward was proportionate to the number of NVDs in the first quarter of 2016 in that ward. Consecutive sampling technique was used to select eligible mothers in each ward.

### Data collection

A thorough literature survey was conducted to identify a suitable instrument to assess client perceptions of institutional postnatal care in Sri Lanka. Though a number of instruments were available for assessment of client satisfaction, instruments assessing client perceived quality of postnatal care were scarce. The possibility of using the instrument used by van Duong et al., to evaluate the client perceived quality of maternity services in rural Vietnam [11] was assessed by an expert panel consisting of three public health specialists. According to the experts, several items in this instrument were not relevant to the institutional postnatal care provision in Sri Lanka. Therefore a new instrument - The Client Perceived Quality of Institutional Postnatal Care (CPQIPNC) questionnaire- was developed by the Principal Investigator (PI), for data collection. It had a total of 23 items divided into three domains: technical care and information, interpersonal care and ward facilities and cleanliness. Each item was rated on a five-point Likert scale, from one to five, and had five statements: “very poor”, “poor”, “neutral”, “good” and “very good”. The scores ranged from one for “very poor” ratings, to five for “very good” ratings. Therefore, the total obtainable score ranged from 23 to 115 (Additional file 1). It was validated by conducting an exploratory and confirmatory factor analysis and had an internal consistency and test retest reliability of 0.94 [18]. In addition, socio demographic information from the mother and information on receipt of services by the mother and the baby that are stipulated in the National guidelines on maternal care [19] were also collected using interviewer administered questionnaires (Additional files 2 and 3).

Interviews were conducted in a pre-identified place in the ward with adequate privacy after all the discharge procedures were completed for each selected mother. As discharges are done twice daily at 12 noon and at 5.00 pm, two interview sessions were done daily, approximately at these times.

Data collection was conducted by six trained pre-intern medical officers via an interviewer administered questionnaire after obtaining the informed written consent from the eligible mothers. Data collectors were supervised by the PI throughout the study and 2% of all questionnaires completed by each data collector was re-administered by the PI to ensure reliability of data collection. Inter-rater reliability was assessed by computing Intraclass correlation coefficient (ICC) for data collectors’ results and PI’s results. The cut off was set at 0.7 [20]. The ICC obtained by data collectors ranged between 0.96–0.99, indicating a satisfactory inter-rater reliability.

### Data analysis

Data analysis was conducted by SPSS version 21. Descriptive statistics were used to describe the basic information of the study sample, services received by them during the postnatal period and their perceived quality.

A binomial logistic regression analysis was carried out to determine the associations of client perceptions, as most of the statistical assumptions were met for binomial regression analysis.

The total score obtained by each participant for the CPQIPNC questionnaire was taken as the dependent variable and was dichotomized into low and high client perceived quality, based on the median value of the total score. Identification of the independent variables was accomplished by reviewing previous literature and expert opinion. The variables included socio demographic factors, pregnancy related factors, institutional factors and services received during the current hospital stay.

Information on independent variables were gathered during interviews of the mothers using questionnaires developed by the PI. They were transformed into categorical variables for further analysis. The independent variables to be included in the analysis were determined by a bivariate analysis. The variables with less than 10 counts in one category were excluded from the analysis. The significance level for selection was taken as *p* < 0.25 [21]. Using the variables selected, a backward stepwise binomial logistic regression was conducted to assess the significant correlates. Goodness of fit of the regression model was assessed by the regression diagnostics (overall percentage of the predictions that were correctly classified by observed outcomes, The Omnibus test, Cox and Snell Square test, Negelkerke R Square test, Hosmer and Lameshow test).

Necessary administrative clearances were obtained and the ethical clearance for the study was obtained from the ethics Review Committee, faculty of Medicine, University of Colombo, Sri Lanka.

## Results

Of the selected mothers, 1265 responded to the questionnaire, giving a response rate of 97.3%. Response rate from individual wards ranged between 76.5–100%. Of the non-respondents, four did not consent for participation (0.3%) and another 31 mothers (2.4%) who consented, had left the hospital before the interview was initiated.

### Socio demographic and pregnancy related details of the participants

Age ranged from 16 to 46 years, while most mothers belonged to the 20–35 years age group (*n* = 1074, 84.9%). More than three fourths have obtained an educational qualification above year 11 (*n* = 1029, 81.3%). A clear majority of the study sample (*n* = 958, 75.7%) was unemployed. Average monthly income showed a wide variation, ranging from Sri Lankan Rupees 3000.00 (17 USD) to 350,000.00 (2000 USD). Close to half of the study sample were primi mothers (*n* = 525, 41.5%) (Table 1).

**Table 1:** Distribution of the study participants by socio demographic characteristics

Socio-demographic characteristic	*N* = 1265	
	N	%
Age in years		
< 20	73	5.8
20–35	1074	84.9
> 35	118	9.3
Ethnicity^a^		
Sinhalese	909	71.9
Moor	184	14.5
Tamil	172	13.6
Religion		
Buddhist	841	66.5
Catholic	112	8.9
Islam	192	15.2
Hindu	120	9.5
Highest level of education		
No schooling	5	0.4
Year 1–5	23	1.8
Year 6–10	208	16.4
Year 11and above	1029	81.3
Occupation		
Unemployed	958	75.7
Temporary employment	101	8.0
Permanent employment	206	16.3
Husbands’ level of education		
No schooling	5	0.4
Year 1–5	17	1.3
Year 6–10	167	13.2
Year 11 and above	1074	84.8
Other^b^	2	0.2
Husbands’ Occupation		
Unemployed	5	0.4
Temporary employment	650	51.4
Permanent employment	608	48.1
Other^b^	2	0.2
Income (Sri Lankan Rupees)		
No income	2	0.2
< 30,000.00	252	19.9
30,000-39,999	344	27.2
40,000-49,999	231	18.3
= > 50,000	436	34.5
Parity (Current)		
Primiparous	525	41.5
Multiparous	740	58.5

^a^The three main ethnic groups in Sri Lanka are Sinhalese, which make up the majority of the population, tamils and moors

^b^These participants did not have a husband

### Mothers’ account on services received by them

Services recommended in the national guidelines to be delivered during the postnatal period following a NVD were received by most of the participants. Majority have initiated breast feeding within 1 h after delivery (*n* = 1184, 93.6%) as recommended, and 99.4% mothers have exclusively breast fed while in the postnatal ward. The technique of breast feeding has been assessed by a health care worker for majority of mothers (*n* = 1249, 98.7%) and corrected where necessary. Least frequently received service was the opportunity to practice kangaroo mother care (KMC) (*n* = 970, 76.7%).

Inquiry was made into the services provided to the mother and the newborn at discharge. All the mothers and 99.8% (*n* = 1263) of the babies have been examined by a medical officer at discharge, and over 95% of the examinees were informed about their examination findings (1235 participants were informed about their examination findings and 1256 were given information following examination of the baby). Privacy has been ensured during 99% of the examinations by covering the examination area (*n* = 1254).

All mothers have received health advices regarding the postnatal period. The main sources of information were nursing officers and midwives (*n* = 1195, 94.5% and *n* = 190, 86.2% respectively). Medical officers have provided health advices to only 750 mothers in the study sample (59.3%) (Table 2).

**Table 2:** Services received by the woman and the baby during the postnatal period

Service received (*N* = 1265)	Frequency	%
Handing over the baby to the woman immediately after the delivery	1104	87.3
Informing the woman about baby’s health after the examination	1209	95.6
Provision of adequate pain relief during the suture of episiotomy	1125	88.9
Provision of refreshment to the woman following delivery	1233	97.5
Practice of kangaroo mother care in the ward	970	76.7
Regular examination of the woman during the postnatal period	1193	94.3
Initiation of breast feeding within 1 h after the delivery	1184	93.6
Receipt of assistance from the staff to initiate breast feeding	1230	97.2
Exclusive breast feeding in the postnatal ward	1258	99.4
Observation of the breast-feeding technique by a health care worker	1249	98.7
Inquire about the baby’s health in the postnatal ward	1260	99.6
Getting assistance to ambulate as soon as possible	1149	90.8
Woman was given the opportunity to keep the baby near her in the postnatal ward	1261	99.7
Received health advices from a doctor	750	59.3
Received health advices from a nursing officer	1195	94.5
Received health advices from a midwife	1090	86.2
Provision of BCG vaccine to the baby before discharge	1261	99.7
Examination of the woman by a medical officer before discharge	1265	100
Conduct of the examination in a covered area	1254	99.1
Presence of a female health care worker if the doctor is male (*N* = 802)	775	96.6
Provision of information about the examination findings	1235	97.6
Examination of the baby before discharge by a medical officer	1263	99.8
Informing the woman about baby’s health after the examination (*n* = 1263)	1256	99.4

### Client perceived quality of institutional postnatal care (CQIPNC)

The total obtainable score of CQIPNC ranged from 23 to 115. The obtainable scores for technical care and information domain, interpersonal care domain and ward facilities and cleanliness domain ranged between 9 - 45, 7–35, and 7–35 respectively.

The total score obtained for the CQIPNC questionnaire in the study ranged from 48 to 115. The median score obtained by the participants for the questionnaire was 108, which was 93.9% of the total obtainable score (IQR- 96-114). Technical care and information domain had a median score of 43 (IQR = 38–45). Interpersonal care domain and ward facilities and cleanliness domain had median scores of 33 (IQR = 30–35) and 32 (IQR = 28–35) respectively (Table 3).

**Table 3:** Median values for each domain of the CPQIPNC questionnaire (*N* = 1265)

Domain (Number of items)	Range	Median	Median as a percentage of the maximum score (%)	IQR
Technical care and Information (9)	16–45	43	95.5	38–45
Interpersonal care (7)	16–35	33	94.3	30–35
Ward facilities and cleanliness (7)	16–35	32	91.4	28–35
Total score	48–115	108	93.9	96–114

Over 90% of the mothers have rated care as ‘good’ or ‘very good’ for all the items included in interpersonal care domain and the technical care and information domain. The ratings reduced somewhat for the items in the ward facilities and cleanliness domain. Only 81.1% have rated the cleanliness of the toilets in the ward as ‘good’ or ‘very good’. The ‘good’ or ‘very good’ ratings percentages for the space and the facilities available were 88.0 and 88.8% respectively (Table 4).

**Table 4:** Percentage of women who perceived each item in the CPQIPNC questionnaire as “good” or “very good” (*N* = 1265)

Domain and Item		Number	Percentage (%)
Interpersonal care			
1	Woman’s perception about the friendliness shown towards her by the HCWs^a^ in the Postnatal Ward	1210	95.7
2	Woman’s perception about the patience shown by the HCWs when she did not cooperate with them	1166	92.2
3	Woman’s perception about the promptness of the attention given by the HCWs when she needed it	1194	94.4
4	Woman’s perception about the availability of pain relief during the postpartum period	1233	97.4
5	Woman’s perception about the way her privacy was respected by the HCWs in the Postnatal Ward	1249	98.8
6	Woman’s perception about the willingness of the health care workers to discuss about her concerns	1155	91.3
7	Woman’s perception about the way health care workers treated her family members	1203	95.1
Technical care and Information			
8	Woman’s perception about the help given for initiation of breast feeding in the labour room	1224	96.8
9	Woman’s perception about the help she received from the health care workers to take care of the baby	1191	94.2
10	Woman’s perception about the help she received from the health care workers to take care of herself	1163	91.9
11	Woman’s perception about the adequacy of information given to take care of the baby	1200	94.8
12	Woman’s perception about the adequacy of information given on proper method of breast feeding	1237	97.8
13	Woman’s perception about the adequacy of information to identify danger signals^b^ following delivery, for the mother & the baby	1163	91.9
14	Woman’s perception about the skills of the HCWs to identify and manage health issues of the baby	1225	96.9
15	Woman’s perception about the skills of the HCWs to identify and manage health issues in relation to her	1219	96.4
16	Woman’s perception about adequacy of information received to clarify any issues she had	1169	92.4
Ward facilities and Cleanliness			
17	Woman’s perception about the Cleanliness of the ward	1222	96.6
18	Woman’s perception about the Cleanliness of the toilets & washrooms	1032	81.1
19	Woman’s perception about adequacy of space in the postnatal ward	1114	88.0
20	Woman’s perception about the availability of adequate facilities in the ward in relation to the number of patients	1124	88.8
21	Woman’s perception about adequacy of delivery beds in the labour room	1235	97.7
22	Woman’s perception about the availability of adequate numbers of HCWs	1232	97.4
23	Woman’s perception about the ability to get some rest in the postnatal ward (without the interferences such as light, noise, ward activities)	1130	89.3

^a^*HCW* Health care worker

^b^Danger signs – instances that requires mother and the baby to return to the hospital/ consult a doctor immediately

### Determinants of client perceived quality of care

For this analysis, the scores obtained for the CPQIPNC were categorized into high perceived quality and low perceived quality, based on the median value obtained for the questionnaire. Thus scores below 108 were categorized as low perceived quality (*n* = 632), and scores equal to or above 108 were categorized as high perceived quality (*n* = 633). The bivariate analysis depicted that participant characteristics such as the age between 20 and 35 years (OR = 1.6, *p* = 0.06), husband’s occupation (OR = 1.2, *p* = 0.08), average monthly income (OR = 0.8, *p* = 0.13), the type of institution used by the mother (OR = 1.4, *p* = 0.004); services such as initiation of breast feeding within 1 h (2.2, *p* = 0.001), informing the mother after examination of the baby (OR = 1.9, *p* = 0.02), provision of adequate pain relief during episiotomy suture (OR = 2.2, *P* < 0.001), giving assistance to practice KMC in the labour room and the ward (OR = 1.4, *p* = 0.02), receiving health advices from doctors (OR = 2.6, *p* < 0.001) and midwives (OR = 3.2, *p* < 0.001), regular examination of the mother (OR = 2.0, *p* = 0.009), getting assistance to initiate breast feeding (OR = 1.9, *p* = 0.12) were significantly associated with high perceptions of quality of care.

Among these variables, only 20–35 age category (aOR = 1.8, *p* = 0.024), teaching and specialized hospitals category (1.6, *p* < 0.001), and services such as initiation of breast feeding within 1 h of delivery (2.1, *p* = 0.009), pain relief during suturing of the episiotomy (2.2, *p* < 0.001), Ability to practice KMC (1.4, *p* = 0.035), Receiving health advices by the doctors (2.1, *p* < 0.001) and PHMs (2.1, *p* < 0.001) were identified as significant correlates via the multivariate analysis (Table 5).

**Table 5:** Results of the logistic regression on factors associated with a positive maternal perception of quality of care received in the institutional postnatal period

Variable	Frequency	Crude OR (*p* value)	aOR (*p* value)
Socio-demographic variables			
Age			
> 35 years	73	1.2 (0.53)	1.3 (0.45)
20–35 years	1074	1.6 (0.06)	1.8 (0.024)
< 20 years	118	1	1
Husbands’ Occupation			
Permanent employment	608	1.2 (0.08)	1.2 (0.08)
Unemployed/ temporary employment	657	1	1
Income			
≥ 40,000 Sri Lankan Rupees	667	0.8 (0.69)	0.8 (0.16)
< 40,000 Sri Lankan Rupees	598	1	1
Institutional characteristics			
Teaching/ Specialized hospitals	994	1.6 (< 0.01)	1.4 (< 0.01)
Base hospitals	271	1	1
Service provided to the woman and the newborn			
Initiation of breast feeding immediately after the delivery			
Yes	1184	2.2 (< 0.01))	2.1 (< 0.01)
No	81	1	1
Provision of adequate pain relief for suture of episiotomy (1264)			
Yes	1125	2.2 (< 0.01)	2.2 (< 0.01)
No	295	1	1
Practicing Kangaroo Mother Care at ward			
Yes	970	1.4 (0.02)	1.4 (0.04)
No	295	1	1
Receipt of health advices from the doctors			
Yes	750	2.6 (< 0.01)	2.1 (< 0.01)
No	515	1	1
Receipt of health advices by the Midwives			
Yes	1090	3.2 (< 0.01)	2.1 (< 0.01)
No	175	1	1
Regular examination of the woman in the postnatal period^a^			
Yes	1193	2.0 (< 0.01)	–
No	72	1	1
Informing about the baby’s health after examination^a^			
Yes	1209	1.9 (0.02)	–
No	56	1	1
Help received from the health staff for breast feeding^a^(1255)			
Yes	1230	1.9 (0.12)	–
No	25	1	1

^a^These variables were removed in the multivariate analysis prior to the final model

The final model explains between 10.7% (Cox & Snell R square) to 14.3% (Negelkerke R Square) of the variation in the client perceived quality. The Omnibus test was statistically significant with a *p* value of less than 0.001. Hosmer and Lameshow test was not significant, indicating that the model was a good fit to the data. The final model of the binary logistic regression correctly classified 52.5% of client perceptions.

## Discussion

Client perceptions of the care received by her or him is increasingly being considered a valid measure of quality of healthcare, despite the subjective nature of the measurement. It gives an immediate feedback to the provider on the services provided. Authorities can use these data in quality improvement processes to identify deficiencies in patient centered care [7].

To the best of authors’ knowledge, this is the first study to assess the client perceived quality of regular postnatal care services provided by public sector specialized institutions in Sri Lanka. This study discovered that mothers’ assessment of the quality of institutional postnatal care was favourable, as observed by the high median scores received for the questionnaire. It also identified that each item of care assessed by the questionnaire was rated favourably by most of the mothers.

Favourable ratings for client satisfaction and client perception surveys is a well-known phenomenon [22] and has been observed across the world [23–25]. A recent review of literature on women’s’ satisfaction with maternal care in developing countries has observed that majority of studies have reported a high level of satisfaction [24]. However, in several studies, clients have rated the quality of institutional maternal care as poor [26, 27] which have been attributed by some authors to poor quality of health delivery systems, client characteristics such as literacy level, cultural diversity, and techniques of assessment of satisfaction.

Client perceived quality is a multi-dimentional concept and is assessed through a number of related domains [28]. During the development of the CPQIPNC tool, three domains were identified, namely, technical care, interpersonal care and ward facilities. These domains have commonly been used to assess client perceptions with institutional care [11, 14]. Among them, “ward facilities and cleanliness” domain was rated less favourably than other two domains by the participant mothers. Items such as cleanliness of the toilets and washrooms, space in the postnatal ward and ability to get adequate rest have obtained lowest scores. Ward facilities and related domains have consistently obtained low scores in client perception and client satisfaction surveys. Wijesinghe [14] and Senerath [29] have also identified the same phenomenon in relation to intra-natal care in Sri Lanka. The same trend has been reported in international studies, where environmental attributes have been rated negatively in comparison to other attributes [28]. This reflects the relative low priority given to the physical environment of the wards by the authorities in general and mothers’ concern about it. A satisfying physical environment will reduce the maternal stress during the immediate postnatal period. Therefore this provides an important feedback to the authorities to ponder when improving care.

Further, attributes such as technical care and interpersonal care may also receive higher scores due to courtesy bias, where inanimate items such as that assessed by ward facilities domain may be rated more objectively, as suggested by studies assessing the biases associated with exit interviews [30, 31]. Further, in the current study, mothers have reported that most of the recommended services were provided to them, which may contribute to high ratings of technical care.

### Assessment of determinants of client-perceived quality of care

Client perceptions may be influenced by many factors besides actual service delivery. Studies have shown that apart from structure, process and outcomes of care, factors such as client characteristics, access to services, financial costs, socio economic and cultural factors may shape the way clients perceive the services [24].

Logistic regression revealed that apart from age, other socio demographic factors were not associated with mothers’ perceptions of the quality of care. Similar findings have been reported by Kambala et al. [32], where socio demographic factors were found to be related to perceived quality of antenatal care but not delivery or postnatal care. Further, literature report that socio demographic associations of client perceptions are not consistent across studies. In addition, most of these associations are non-modifiable, limiting their value in improving care to meet patients’ expectations [33].

Awareness on service related and facility related factors that may influence mothers’ perceptions on the other hand, plays a major role in improving care. The current study found that certain services, such as initiation of breast feeding within 1 h of delivery, pain relief during suturing of the episiotomy, ability to practice KMC, receiving health advices by the doctors and midwives were associated with higher ratings of quality of care provided to mothers and the babies. These services may help to create a favourable opinion of the postnatal care in the mother, leading to high perceived quality.

Initiation of breast feeding and skin to skin care immediately after the delivery promotes the bonding between mother and the newborn has been known to improve the maternal perceptions and satisfaction with care [34, 35]. Practice of Kangaroo Mother Care also improves bonding through skin to skin care, leading to positive perceptions about quality of care.

Experience of intense pain during labour by the mother has constantly been associated with negative perceptions about labour [36]. Therefore, it is reasonable to expect that mothers who received adequate pain relief during suture of episiotomy would perceive care more positively compared to mothers who did not, as demonstrated by the current study.

Previous studies have reported that mothers expect to receive adequate information on important aspects such as breast feeding, child-care and behaviour during the postnatal period [37]. Positive perceptions of care by the mothers who receive information as expected is evident in many literature on maternal satisfaction [24]. Consistently, the current study revealed that while all mothers have received health advices from either midwives, nurses or doctors, those who received advices from doctors and midwives have perceived care more positively than who did not.

In addition to these services, mothers who received services from teaching and specialized hospitals have rated the quality more favourably than the mothers in the base hospitals. “Good physical environment and efficient management” has been stated as significant predictors of positive assessment of care by mothers [24]. Teaching hospitals and specialized hospitals have better facilities and resources than base hospitals and is capable of better management and care than base hospitals. This is consistent with literature that higher-level institutions are associated with more positive maternal perceptions of care, possibly due to better facilities [28].

## Limitations of the study

Our study had following limitations. Firstly, it assessed the perceptions of the mothers who attend specialized institutions only. The perceptions of the mothers who attend non-specialized institutions may be quite different to these findings. Secondly the effect of courtesy bias that is introduced when information is collected through exit interviews may have led to an over estimation of the results. However, use of interviewers who were not involved in patient care and ensuring privacy during the interviews were aimed to overcome this issue.

## Conclusions and recommendations

This study presents a cross sectional depiction of postnatal mothers’ perceptions of the care received by them. According to the postnatal mothers’ perceptions, the quality of institutional postnatal care in specialized institutions in Colombo district of Sri Lanka is commendable. However, the ward facilities and environment domain has obtained lower ratings compared to technical and interpersonal care domains. Services such as initiation of breast feeding immediately following delivery, practice of KMC in the ward, getting health advices from doctors or midwives and provision of adequate pain relief during episiotomy suture, and obtaining services from higher level institutions were associated with positive client perceptions. These findings will be useful for quality improvement, and to provide more patient centred care. However, quality assessment should be a regular process which should be an integral component of health care delivery systems. Therefore, it is recommended that this assessment is conducted regularly to get the most updated feedback.

## Supplementary information

**Additional file 1.** Tool to assess client perceived quality of institutional postnatal care- CPQIPNC Questionnaire.

**Additional file 2.** Questionnaire to assess socio-demographic details of the mothers.

**Additional file 3.** Checklist to assess the provision of regular postnatal care following normal vaginal delivery.

## Data Availability

The datasets used and/or analysed during the current study are available from the corresponding author on request.

## Abbreviations

aOR: Adjusted Odds Ratio
CPQIPNC: Client Perceived Quality of Institutional Postnatal Care
HCW: Health care worker
ICC: Intra-class Correlation Coefficient
IQR: Inter Quartile Range
KMC: Kangaroo mother care
MNH: Maternal and Newborn Health
NVD: Normal vaginal delivery
OR: Odds Ratio
PI: Principal Investigator
SPSS: Statistical Package for the Social Sciences
USD: United States Dollars
