# Supplementary material for: Client perceived quality of the postnatal care provided by public sector specialized care institutions following a normal vaginal delivery in Sri Lanka: a cross sectional study
Source: BMC Pregnancy Childbirth. 2019 Dec 9;19:485. doi: 10.1186/s12884-019-2645-4 (PMC6902491; doi:10.1186/s12884-019-2645-4)
Supplement: Supplementary file 1 — Additional file 1. Tool to assess client perceived quality of institutional postnatal care- CPQIPNC Questionnaire. [file 12884_2019_2645_MOESM1_ESM.docx]

**Tool to assess client perceived quality of institutional postnatal care- CPQIPNC Questionnaire**

1-Very poor; 2- Poor; 3- Neutral; 4- Good; 5- Very good

|  | **ITEMS** | 1 | 2 | 3 | 4 | 5 |
| --- | --- | --- | --- | --- | --- | --- |
| 1 | Your perception about the friendliness shown towards you by the HCWs in the Postnatal Ward |  |  |  |  |  |
| 2 | Your perception about the patience shown towards you when you did not cooperate with HCWs |  |  |  |  |  |
| 3 | Your perception about the promptness of the attention given by the HCWs when you needed it |  |  |  |  |  |
| 4 | Your perception about the availability of pain relief during the postpartum period |  |  |  |  |  |
| 5 | Your perception about the way your privacy was respected by the HCWs in the PNW |  |  |  |  |  |
| 6 | Your perception about the willingness of the health care workers to discuss about your concerns |  |  |  |  |  |
| 7 | Your perception about the way health care workers treated your family members |  |  |  |  |  |
| 8 | Your perception about the help given for the initiation of breast feeding in the labour room |  |  |  |  |  |
| 9 | Your perception about the help you received from the health care workers to take care of your baby |  |  |  |  |  |
| 10 | Your perception about the help you received from the health care workers to take care of yourself (ex: maintaining your cleanliness) |  |  |  |  |  |
| 11 | Your perception about the adequacy of information given to you on taking care of the baby |  |  |  |  |  |
| 12 | Your perception about the adequacy of information given on proper method of breast feeding |  |  |  |  |  |
| 13 | Your perception about the adequacy of information to identify danger signals following delivery, for the mother & the baby |  |  |  |  |  |
| 14 | Your perception about the skills of the HCWs to identify and manage health issues of your baby |  |  |  |  |  |
| 15 | Your perception about the skills of the HCWs to identify and manage health issues in relation to you |  |  |  |  |  |
| 16 | Your perception about adequacy of information received to clarify any issues you had |  |  |  |  |  |
| 17 | Your perception about the Cleanliness of the ward |  |  |  |  |  |
| 18 | Your perception about the Cleanliness of the toilets & washrooms |  |  |  |  |  |
| 19 | Your perception about adequacy of space in the postnatal ward |  |  |  |  |  |
| 20 | Your perception about the availability of adequate facilities in the ward in relation to the number of patients |  |  |  |  |  |
| 21 | Your perception about adequacy of delivery beds in the labour room |  |  |  |  |  |
| 22 | Your perception about the availability of adequate numbers of HCWs to assist you |  |  |  |  |  |
| 23 | Your perception about the ability to get some rest in the postnatal ward (without the interferences such as light, noise, ward activities) |  |  |  |  |  |
