# Supplementary material for: Client perceived quality of the postnatal care provided by public sector specialized care institutions following a normal vaginal delivery in Sri Lanka: a cross sectional study
Source: BMC Pregnancy Childbirth. 2019 Dec 9;19:485. doi: 10.1186/s12884-019-2645-4 (PMC6902491; doi:10.1186/s12884-019-2645-4)
Supplement: Supplementary file 2 — Additional file 2. Questionnaire to assess socio-demographic details of the mothers. [file 12884_2019_2645_MOESM2_ESM.docx]

| Date: |  |
| --- | --- |
| Serial No: |  |

**QUESTIONNAIRE TO ASSESS SOCIO-DEMOGRAPHIC DETAILS OF THE MOTHERS**

Please put an **X** in the appropriate box or write the answer in the given space

**[A] Socio Demographic Details:**

| Name of the Hospital : | | | |
| --- | --- | --- | --- |
| Unit : | | | |
| Date of Birth : | | | |
|  |  | |  |
| Ethnicity | Sinhala | |  |
|  | Tamil | |  |
|  | Moor | |  |
|  | Other (Specify) | | |
|  |  | |  |
| Religion | Buddhism | |  |
|  | Christian | |  |
|  | Hindu | |  |
|  | Islam | |  |
|  | Other (Specify) | | |
|  |  |  | |
| What is the highest level of education achieved by you :  (Highest grade/ examination completed?) | | | |
| What is your occupation : | | | |
| What is your husband’s/partner’s occupation : | | | |
| What is the highest level of education achieved by your husband/partner :  (Highest grade/ examination completed?) | | | |
| What is your family’s average monthly income? | | | |
| What is the distance to this hospital from your home? | | | |
| Why did you choose this hospital for your delivery? | | | |
| Did you face any unpleasant situations in the hospital after you got admitted for this delivery? If so, what is it? | | | |

**[B] Pregnancy Related Information**

B1) which pregnancy is this pregnancy? ……………………………………..

B4) information on previous pregnancies:

|  | Outcome (Live/Still birth/ Miscarriage) | Hospital where delivery took place | Any unpleasant situations during that admission | satisfaction with the services received at that hospital  (satisfied/ not) |
| --- | --- | --- | --- | --- |
| P1 |  |  |  |  |
| P2 |  |  |  |  |
| P3 |  |  |  |  |
| P4 |  |  |  |  |
| P5 |  |  |  |  |
|  |  |  |  |  |
|  |  |  |  |  |
