# Supplementary material for: Client perceived quality of the postnatal care provided by public sector specialized care institutions following a normal vaginal delivery in Sri Lanka: a cross sectional study
Source: BMC Pregnancy Childbirth. 2019 Dec 9;19:485. doi: 10.1186/s12884-019-2645-4 (PMC6902491; doi:10.1186/s12884-019-2645-4)
Supplement: Supplementary file 3 — Additional file 3. Checklist to assess the provision of regular postnatal care following normal vaginal delivery. [file 12884_2019_2645_MOESM3_ESM.docx]

**Checklist to assess the provision of regular postnatal care following normal vaginal delivery**

Services you have received during and after delivery have been inquired by this questionnaire. Please answer the questions based on the services received by **you**. When you can’t provide an answer for a question asked (for example when you are not sure or when you have not seen whether you or the baby received the service stipulated by the question) please answer as “not sure”.

**Care Received in the labour room**

| Was the baby delivered onto your abdomen? | | | **Yes** | | **No** | **Not sure** |
| --- | --- | --- | --- | --- | --- | --- |
|  | | |  | |  |  |
| Was the baby given to you immediately after delivery? | | | **Yes** | | **No** | **Not sure** |
|  | | |  | |  |  |
| Were you informed about the baby’s condition after initial examination? | | | **Yes** | | **No** | **Not sure** |
|  | | |  | |  |  |
| How soon did you start breast feeding after delivery? | | |  | |  |  |
| Within one hour of birth | After one hour of birth | In the postnatal ward | | other | | |
|  | | |  | |  |  |
| Did you get support of the healthcare workers (PHM/ Nurses) to initiate breast feeding? | | | **Yes** | | **No** | **Not sure** |
|  | | |  | |  |  |
| Was baby’s weight checked before or after initiation of breast feeding | | | **Yes** | | **No** | **Not sure** |
|  | | |  | |  |  |
| Were you given adequate pain relief during the suture of the episiotomy | | | **Yes** | | **No** | **Not sure** |
|  | | |  | |  |  |
| Did you practice skin to skin contact (Baby is put on the bare skin of the chest of the mother) in the Labour room | | | **Yes** | | **No** | **Not sure** |
|  | | |  | |  |  |
| Were your health status checked regularly in the labour room following delivery? | | | **Yes** | | **No** | **Not sure** |
|  | | |  | |  |  |
| Were you cleaned after the delivery and put on clean clothes | | | **Yes** | | **No** | **Not sure** |
|  | | |  | |  |  |
| Was your bed linen changed whenever it got soiled (Got contaminated with blood) | | | **Yes** | | **No** | **Not sure** |
|  | | |  | |  |  |
| Were you informed about the disc number placed on yours’ and baby’s hand? | | | **Yes** | | **No** | **Not sure** |
|  | | |  | |  |  |
| Were you offered refreshment after delivery? | | | **Yes** | | **No** | **Not sure** |

**Services delivered in the postnatal ward**

| Did you exclusively breast feed your baby while in the ward? | | | | | **Yes** | | **No** | | **Not sure** | |
| --- | --- | --- | --- | --- | --- | --- | --- | --- | --- | --- |
|  | | | | |  | |  | | |  |
| If not, What was the reason for not breast feeding exclusively? | | | | |  | |  | | |  |
| …………………………………………………………… | | | | | | | | | | |
| What else did you give? | | | | |  | |  | |  | |
| ……………………………………………………………. | | | | |  | |  | |  | |
| Did a PHM/Nurse assess your technique of breast feeding? | | | | | **Yes** | | **No** | | **Not sure** | |
|  | | | | |  | |  | |  | |
| If she did, what were you told about the technique? | | | | |  | |  | |  | |
| Method is correct | Method is not correct | Said nothing | | | | Other | | | | |
|  | | | | |  | |  | |  | |
| If the technique was not correct, did you get their support to correct the technique? | | | | | **Yes** | | **No** | | **Not sure** | |
|  | | | | |  | |  | |  | |
| Did you practice skin to skin contact in the PNW | | | | | **Yes** | | **No** | | **Not sure** | |
|  | | | | |  | |  | |  | |
| Was your baby bathed before discharge? | | | | | **Yes** | | **No** | | **Not sure** | |
|  | | | | |  | |  | |  | |
| If so, how many hours after the delivery was the baby bathed? (Give a rough time) …………………………………………. | | | | |  | |  | |  | |
|  | | | | |  | |  | |  | |
| Did postnatal wad staff check if the baby passed faeces before discharge | | | | | **Yes** | | **No** | | **Not sure** | |
|  | | | | |  | |  | |  | |
| Did they check if baby passed urine well | | | | | **Yes** | | **No** | | **Not sure** | |
|  | | | | |  | |  | |  | |
| Was your blood pressure checked when you were in postnatal ward | | | | | **Yes** | | **No** | | **Not sure** | |
|  | | | | |  | |  | |  | |
| Were you assessed regularly for presence of vaginal bleeding while in the postnatal ward | | | | | **Yes** | | **No** | | **Not sure** | |
|  | | | | |  | |  | |  | |
| Was your bed linen changed whenever it got soiled (Got contaminated with blood) | | | | | **Yes** | | **No** | | **Not sure** | |
|  | | | | |  | |  | |  | |
| Were you encouraged to mobilize while in the PNW | | | | | **Yes** | | **No** | | **Not sure** | |
|  | | | | |  | |  | |  | |
| Was the baby kept with you in your bed while in the ward? | | | | | **Yes** | | **No** | | **Not sure** | |
|  | | | | |  | |  | |  | |
| If the baby was kept in a cot, was the cot placed in a place where you can reach out easily from the bed? | | | | | **Yes** | | **No** | | **Not sure** | |
|  | | | | |  | |  | |  | |
| Were you advised on following before discharge: | | | | |  | |  | |  | |
|  | | | | |  | |  | |  | |
| selecting an appropriate family planning method | | | | | **Yes** | | **No** | | **Not sure** | |
|  | | | | |  | |  | |  | |
| Exclusive Breast feeding | | | | | **Yes** | | **No** | | **Not sure** | |
|  | | | | |  | |  | |  | |
| importance of proper nutrition during the post-partum period | | | | | **Yes** | | **No** | | **Not sure** | |
|  | | | | |  | |  | |  | |
| how to care for the newborn | | | | | **Yes** | | **No** | | **Not sure** | |
|  | | | | |  | |  | |  | |
| Postpartum hygiene (hand washing, using clean pads) | | | | | **Yes** | | **No** | | **Not sure** | |
|  | | | | |  | |  | |  | |
| Vaccinating the baby | | | | | **Yes** | | **No** | | **Not sure** | |
|  | | | | |  | |  | |  | |
|  | | | | |  | |  | |  | |
| Who gave you these advises | | | | |  | |  | |  | |
|  | | | | |  | |  | |  | |
| Medical Officer | | | | | **Yes** | | **No** | |  | |
|  | | | | |  | |  | |  | |
| Nursing Officer | | | | | **Yes** | | **No** | |  | |
|  | | | | |  | |  | |  | |
| Public Health Midwife | | | | | **Yes** | | **No** | |  | |
|  | | | | |  | |  | |  | |
| Were you taught about the following danger signs of the newborn before discharge? | | | | | | | | | | |
|  | | | | | | | | | | |
| Fast or slow breathing | | | | | **Yes** | | **No** | | **Not sure** | |
|  | | | | |  | |  | |  | |
| Convulsions | | | | | **Yes** | | **No** | | **Not sure** | |
|  | | | | |  | |  | |  | |
| Bluish discolouration of the body | | | | | **Yes** | | **No** | | **Not sure** | |
|  | | | | |  | |  | |  | |
| Fever | | | | | **Yes** | | **No** | | **Not sure** | |
|  | | | | |  | |  | |  | |
| Cold body | | | | | **Yes** | | **No** | | **Not sure** | |
|  | | | | |  | |  | |  | |
| Bleeding from umbilical stump | | | | | **Yes** | | **No** | | **Not sure** | |
|  | | | | |  | |  | |  | |
| Yellow discoloration of the body | | | | | **Yes** | | **No** | | **Not sure** | |
|  | | | | |  | |  | |  | |
| Umbilicus draining pus or umbilical redness extending to skin | | | | | **Yes** | | **No** | | **Not sure** | |
|  | | | | |  | |  | |  | |
| skin pustules | | | | | **Yes** | | **No** | | **Not sure** | |
|  | | | | | | | | | | |
| Were you taught about following danger signs of the mother before discharge? | | | | | | | | | | |
|  | | | | | | | | | | |
| Severe headache | | | | | **Yes** | | **No** | | **Not sure** | |
|  | | | | |  | |  | |  | |
| Burning pain or tightness in the chest or upper abdomen | | | | | **Yes** | | **No** | | **Not sure** | |
|  | | | | |  | |  | |  | |
| Blurring of vision or visual disturbances | | | | | **Yes** | | **No** | | **Not sure** | |
|  | | | | |  | |  | |  | |
| Shortness of breathing | | | | | **Yes** | | **No** | | **Not sure** | |
|  | | | | |  | |  | |  | |
| Increased vaginal bleeding or bleeding with clots | | | | | **Yes** | | **No** | | **Not sure** | |
|  | | | | |  | |  | |  | |
| Pain in the calf muscles which increased with walking | | | | | **Yes** | | **No** | | **Not sure** | |
|  | | | | |  | |  | |  | |
| Foul smelling/ excessive or purulent vaginal discharge | | | | | **Yes** | | **No** | | **Not sure** | |
|  | | | | |  | |  | |  | |
| Episiotomy wound – gaping, infected, abscess formation or severe pain | | | | | **Yes** | | **No** | | **Not sure** | |
|  | | | | |  | |  | |  | |
| Fever | | | | | **Yes** | | **No** | | **Not sure** | |
|  | | | | |  | |  | |  | |
| Were you given Vitamin A megadose before discharge? | | | | | **Yes** | | **No** | | **Not sure** | |
|  | | | | |  | |  | |  | |
| Were you examined by a doctor before discharge? | | | | | **Yes** | | **No** | | **Not sure** | |
|  | | | | |  | |  | |  | |
| Was a VE carried out during the examination? | | | | | **Yes** | | **No** | | **Not sure** | |
|  | | | | |  | |  | |  | |
| Were the screens drawn during examination? | | | | | **Yes** | | **No** | | **Not sure** | |
|  | | | | |  | |  | |  | |
| Were a female hcw present during examination if the examining doctor was a male doctor? | | | **Yes** | **No** | | **Not sure** | | **Doctor was female** | | |
|  | | | | |  | |  | |  | |
| Were other mothers present in the room/examination area during examination? | | | | | **Yes** | | **No** | | **Not sure** | |
|  | | | | |  | |  | |  | |
| Were you informed about your health status after that examination? | | | | | **Yes** | | **No** | | **Not sure** | |
|  | | | | |  | |  | |  | |
| Were your baby examined by a doctor before discharge in the PNW? | | | | | **Yes** | | **No** | | **Not sure** | |
|  | | | | |  | |  | |  | |
| Were you informed about the baby’s health status after that examination? | | | | | **Yes** | | **No** | | **Not sure** | |
|  | | | | |  | |  | |  | |
| Was the baby vaccinated with BCG before discharge? | | | | | **Yes** | | **No** | | **Not sure** | |
|  | | | | |  | |  | |  | |
| Were you advised to inform the area PHM as soon as you go home? | | | | | **Yes** | | **No** | | **Not sure** | |
|  | | | | |  | |  | |  | |
| Hve you decided on the family planning method you are going to use? | | | | | **Yes** | | **No** | | **Not sure** | |
|  | | | | |  | |  | |  | |
| If so, what is it  ………………………………./ have decided not to use | | | | |  | |  | |  | |
